# Comparison of Systemic and Mucosal Immunization with Helper-Dependent Adenoviruses for Vaccination against Mucosal Challenge with SHIV

**DOI:** 10.1371/journal.pone.0067574

**Published:** 2013-07-03

**Authors:** Eric A. Weaver, Pramod N. Nehete, Bharti P. Nehete, Guojun Yang, Stephanie J. Buchl, Patrick W. Hanley, Donna Palmer, David C. Montefiori, Guido Ferrari, Philip Ng, K. Jagannadha Sastry, Michael A. Barry

**Affiliations:** 1 Department of Internal Medicine, Division of Infectious Diseases, Translational Immunovirology Program, Department of Immunology, Mayo Clinic, Rochester, Minnesota, United States of America; 2 Department of Molecular Medicine, Mayo Clinic, Rochester, Minnesota, United States of America; 3 Department of Veterinary Sciences, The University of Texas M.D. Anderson Cancer Center, Houston, Texas, United States of America; 4 Department of Immunology, The University of Texas M.D. Anderson Cancer Center, Houston, Texas, United States of America; 5 Department of Molecular and Human Genetics, Baylor College of Medicine, Houston, Texas, United States of America; 6 Duke University Medical Center, Department of Surgery, Durham, North Carolina, United States of America; University of Alabama, United States of America

## Abstract

Most HIV-1 infections are thought to occur at mucosal surfaces during sexual contact. It has been hypothesized that vaccines delivered at mucosal surfaces may mediate better protection against HIV-1 than vaccines that are delivered systemically. To test this, rhesus macaques were vaccinated by intramuscular (i.m.) or intravaginal (ivag.) routes with helper-dependent adenoviral (HD-Ad) vectors expressing HIV-1 envelope. Macaques were first immunized intranasally with species C Ad serotype 5 (Ad5) prior to serotype-switching with species C HD-Ad6, Ad1, Ad5, and Ad2 vectors expressing env followed by rectal challenge with CCR5-tropic SHIV-SF162P3. Vaccination by the systemic route generated stronger systemic CD8 T cell responses in PBMC, but weaker mucosal responses. Conversely, mucosal immunization generated stronger CD4 T cell central memory (Tcm) responses in the colon. Intramuscular immunization generated higher levels of env-binding antibodies, but neither produced neutralizing or cytotoxic antibodies. After mucosal SHIV challenge, both groups controlled SHIV better than control animals. However, more animals in the ivag. group had lower viral set points than in in the i.m. group. These data suggest mucosal vaccination may have improve protection against sexually-transmitted HIV. These data also demonstrate that helper-dependent Ad vaccines can mediate robust vaccine responses in the face of prior immunity to Ad5 and during four rounds of adenovirus vaccination.

## Introduction

Gene-based vaccines are one approach to vaccinate against HIV-1 wherein viral genes are expressed from an expression vector to stimulate the immune system (reviewed in [1]). Adenoviruses (Ads) are one of a number of gene delivery vectors that are being investigated as gene-based vaccines for HIV-1 [2]–[11].

The earliest work with adenovirus vaccines utilized replication-competent Ad (RC-Ad) vectors with intact E1 early genes and small HIV gene insertions into the viral genome [2]. These vectors have the advantage of up to 10,000-fold vector amplification in infected cells to increase antigen gene copy number and antigen protein production. However, one limitation is the risk of frank adenovirus infection with the use of these vectors. Most Ad HIV vaccines have instead utilized first generation Ad (FG-Ad) vectors that are rendered replication-defective due to deletion of the E1 gene [3]–[5]. These vectors are also usually deleted for the Ad E3 immune evasion genes to make space for larger transgene insertions.

More recently, helper-dependent Ad (HD-Ad) vaccines have been tested as HIV vaccines [10], [12]. Unlike FG-Ad vectors, all Ad genes are deleted from HD-Ad to eliminate expression of potentially inflammatory and immunogenic adenovirus proteins. In gene therapy tests, HD-Ad vectors have been shown to be less immunogenic, have improved safety, and mediate extended expression of transgene products relative to FG vectors [13]–[16].

We performed the first head to head comparison of RC-Ad5, FG-Ad5, and HD-Ad5 vector vaccines [12]. Direct comparison of the three vector platforms in mice demonstrated that RC-Ad5 and HD-Ad5 induced significantly higher immune responses than FG-Ad after i.m. or i.v. injection. This work also showed that HD-Ad5 with all Ad genes removed produced less liver damage than the other vectors and lower anti-Ad T cell responses [12].

Based on these data, HD-Ad vectors were tested as vaccines in Ad5-immunized macaques by serotype-switching three species C HD-Ads expressing HIV-1 env for three rounds of immunization in Ad5-immune macaques [10], [12]. This work demonstrated that serotype-switching with three rounds of HD-Ad6, HD-Ad1, and HD-Ad2 generates significantly higher antibody responses than three rounds of immunization with the single-serotype HD-Ad5 [12]. After mucosal rectal SHIV-SF162P3 challenge, both vaccinated groups controlled viremia significantly better than control animals [10]. However, serotype-switching mediated significantly lower peak viremia than immunization with only the single serotype HD-Ad5 [10]. This is interesting given that these HD-Ad5-immunized animals still mediated protection in the face of several rounds of prior immunization with Ad5.

Since greater than 90% of HIV-1 infections occur at the mucosal surface it is understood that mucosal immune responses may be essential for prophylactic vaccination [17]. However, there is disagreement in which routes of vaccination might induce optimal mucosal immune responses to repel HIV. One hypothesis is that the best way to induce the highest levels and affinities of mucosal immune responses is to deliver the vaccines to mucosal tissues. An alternate hypothesis is that strong systemic immune responses will cross-over into mucosa to repel virus at this site of entry. Both arguments are valid and there is conflicting evidence for both hypotheses in different vaccine-infection systems.

In this study, we compared systemic intramuscular (i.m.) vaccination with mucosal intravaginal (ivag.) vaccination with HD-Ad vectors in the rhesus macaque model. Macaques were first immunized with FG-Ad5. Groups of four macaques were then immunized by the i.m. or ivag. route with HD-Ad6-Env expressing the gp140CF immunogen of HIV-1 JRFL. They were then boosted by the same routes with HD-Ad1-Env, HD-Ad5-Env, and HD-Ad2-Env at 3, 8 and 9 week intervals and were then challenged by the rectal route with 1000 TCID50 of CCR5-tropic SHIV-SF162P3 at week 32 to test if either route of vaccination mediated better immune correlates or protection after mucosal challenge.

## Results

### Comparison of Systemic and Mucosal HD-Ad Vaccination in Ad5-immunized Macaques

We previously compared serotype switching HD-Ad6, 1, and 2 with single serotype vaccination with HD-Ad5 [10], [12]. Groups of four macaques were immunized with HD-Ads expressing only HIV-1 envelope immunogen gp140CF immunogen from the JRFL strain of the virus. This HIV env immunogen fuses gp120 and gp41 by deletion of their furin cleavage site and is also deleted for gp160's transmembrane domain to enable secretion from cells [10], [12]. The animals were challenged with SHIV-SF162P3 expressing the SF162P3 envelope that evolved during three passages in monkeys [18]–[22]. ClustalW alignment of the gp140 domains of JRFL and SF162P3 reveals they have 88% identity in amino acid sequence (Figure S1). Under these conditions, 10 of 11 animals became viremic after rectal challenge with 1000 TCID50 of SHIV-SF162P3 and were available for virologic comparison. Comparison of these small groups of infected animals demonstrated statistically significant reductions in peak viremia between the groups [23]. Based on this prior study in which statistical differences were observed with the small set of animals, the current study was initiated utilizing three groups of four macaques to explore the role of vaccine route in protection against mucosal challenge.

**Figure 1:**
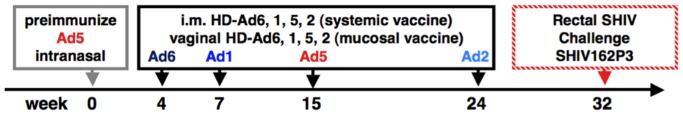
Schedule of Vaccination and SHIV Challenge.

Twelve female macaques were immunized intranasally with 1×10^11^ virus particles (vp) of adenovirus serotype 5 (Ad5) expressing β-galactosidase to initiate immune responses to Ad5 just prior to vaccination with the species HD-Ad vectors (Fig. 1). One group of four macaques was held as controls. One group of four macaques was immunized by i.m. injection with 1×10^11^ vp of HD-Ad6 expressing the JRFL gp140CF immunogen (HD-Ad6-Env) as a systemic vaccine. Another group was immunized by intravaginal (ivag.) lavage with 1×10^11^ vp of HD-Ad6-Env as a mucosal vaccine. Three weeks later, both vaccine groups were boosted by the same routes, this time by switching the serotype to HD-Ad1-Env with the intent to avoid previously induced anti-Ad5 and anti-Ad6 immune responses.

### Systemic T Cell Responses in PBMCs

**Figure 2:**
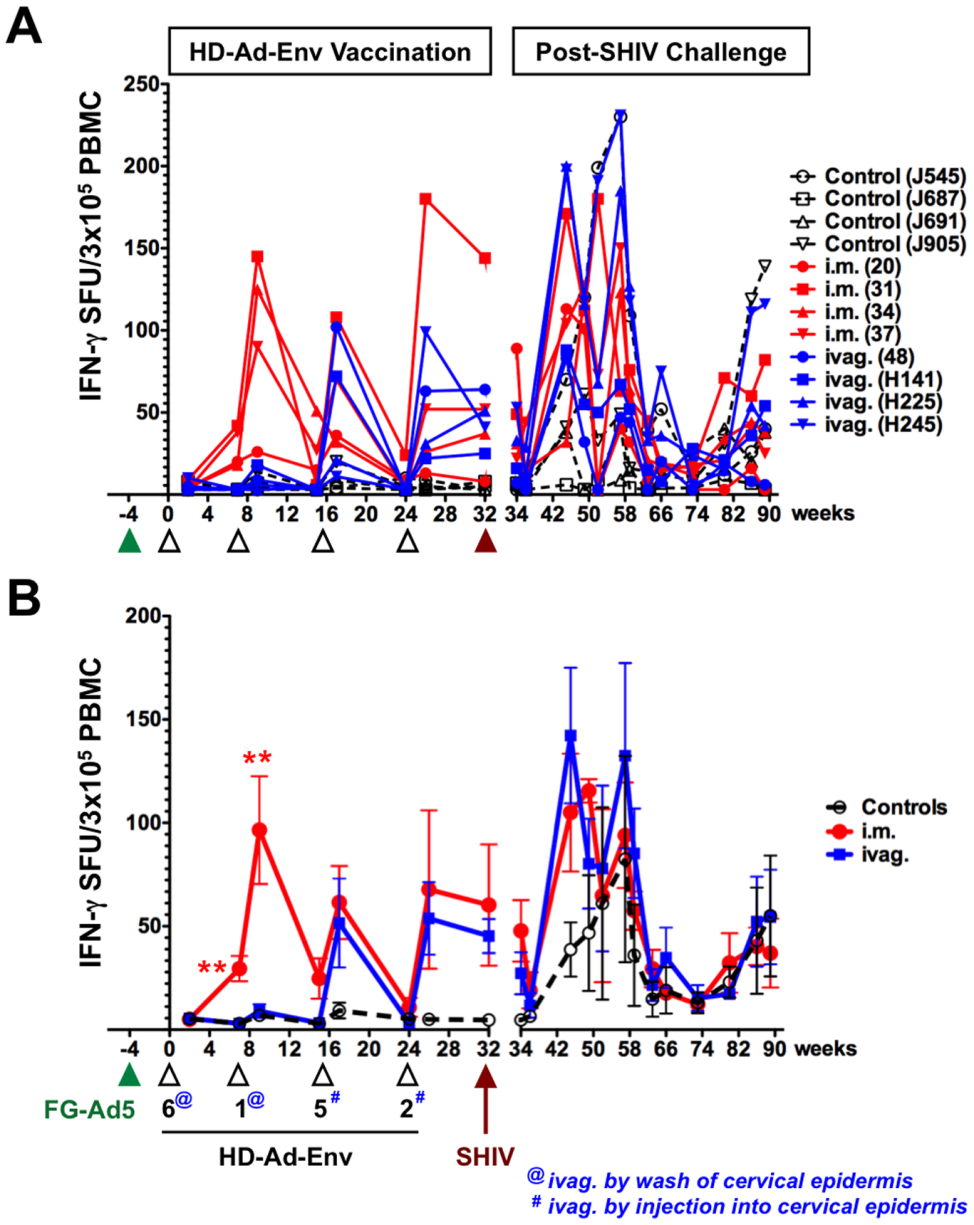
IFN-γ Secreting Cells from PBMCs. PBMCs in ELISPOT plates were stimulated with 3 pools of 50 to 70 peptides spanning the gp140 region of SF162 envelope for 36 h and IFN-γ secreting spots were detected and counted. Responses in terms of IFN-γ spot forming units (SFU) for 10^5^ total input cells were determined for individual monkeys after subtracting background values of cells cultured in the medium. **A**) IFN-γ secreting cells from individual animals. **B**) Mean IFN-γ secreting cells from each group.

After two rounds of immunization, T cell responses mediated by the vaccines were measured from PBMCs. PBMCs were stimulated with the 15-mer peptide set for SF162P3 (NIH AIDS Reagent Program) spanning the gp140 region expressed by the JRFL gp140. 1×10^5^ PBMCs were stimulated separately with 3 pools of 50 to 70 peptides spanning the gp140 region and IFN-γ responses were detected by ELISPOT (Fig. 2). Since no notable differences were observed between the 3 peptide pools and that the pooling were arbitrary mixes, data is shown for the sum of IFN-γ spot forming units (SFU) for 3×10^5^ total input cells for simplicity (Fig. 2).

**Figure 3:**
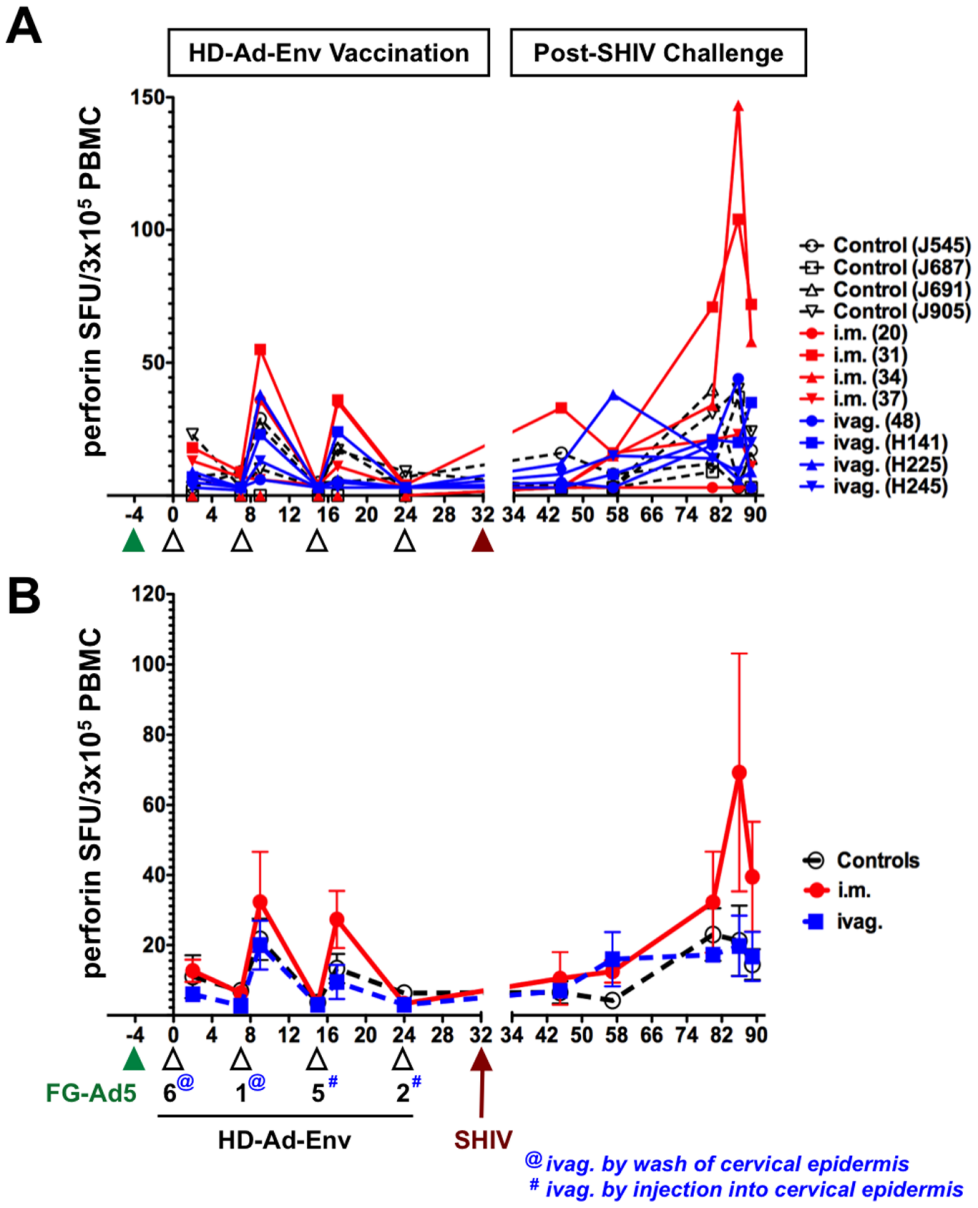
Perforin Secreting Cells from PBMCs. PBMCs were analyzed by ELISPOT as in Fig. 2, but in this case were stained for perforin production. **A**) Perforin secreting cells from individual animals. **B**) Mean perforin secreting cells from each group.

Initial prime-boost with HD-Ad6 and HD-Ad1-Env by the i.m. route generated 20 to 150 IFN-γ SFUs per 3×10^5^ PBMCs (Fig. 2). In contrast, intravaginal lavage with the same vaccines generated weaker responses of 15 or less SFUs. These responses were significantly weaker than those by the i.m. group (p<0.01 by ANOVA). ELISPOT for perforin-secreting PBMCs showed weaker responses that were not different between controls, i.m., and ivag. groups (Fig. 3).

### Modification of Vaginal Immunization Method

The weak T cell responses after vaginal lavage with HD-Ad suggested that this method might be ineffective and endanger the over-all comparison of vaccine delivery to mucosa versus muscle. Given this, and previous observations of improved adenovirus transduction after direct needle injection into the cervical epithelium, the last two mucosal immunizations were performed by injection into this site (note: @ vs. # in Figs. 2 and 3). This altered method would be unlikely to go systemic and would restrict Ad exposure to mucosal tissues.

When this change in vaginal vaccine delivery was used for HD-Ad5-Env and HD-Ad2, they mediated substantially increased IFN-γ responses in PBMCs that approached the magnitude observed in the i.m. group (Fig. 2).

### Vaccine-induced Systemic Cytokine Responses

**Figure 4:**
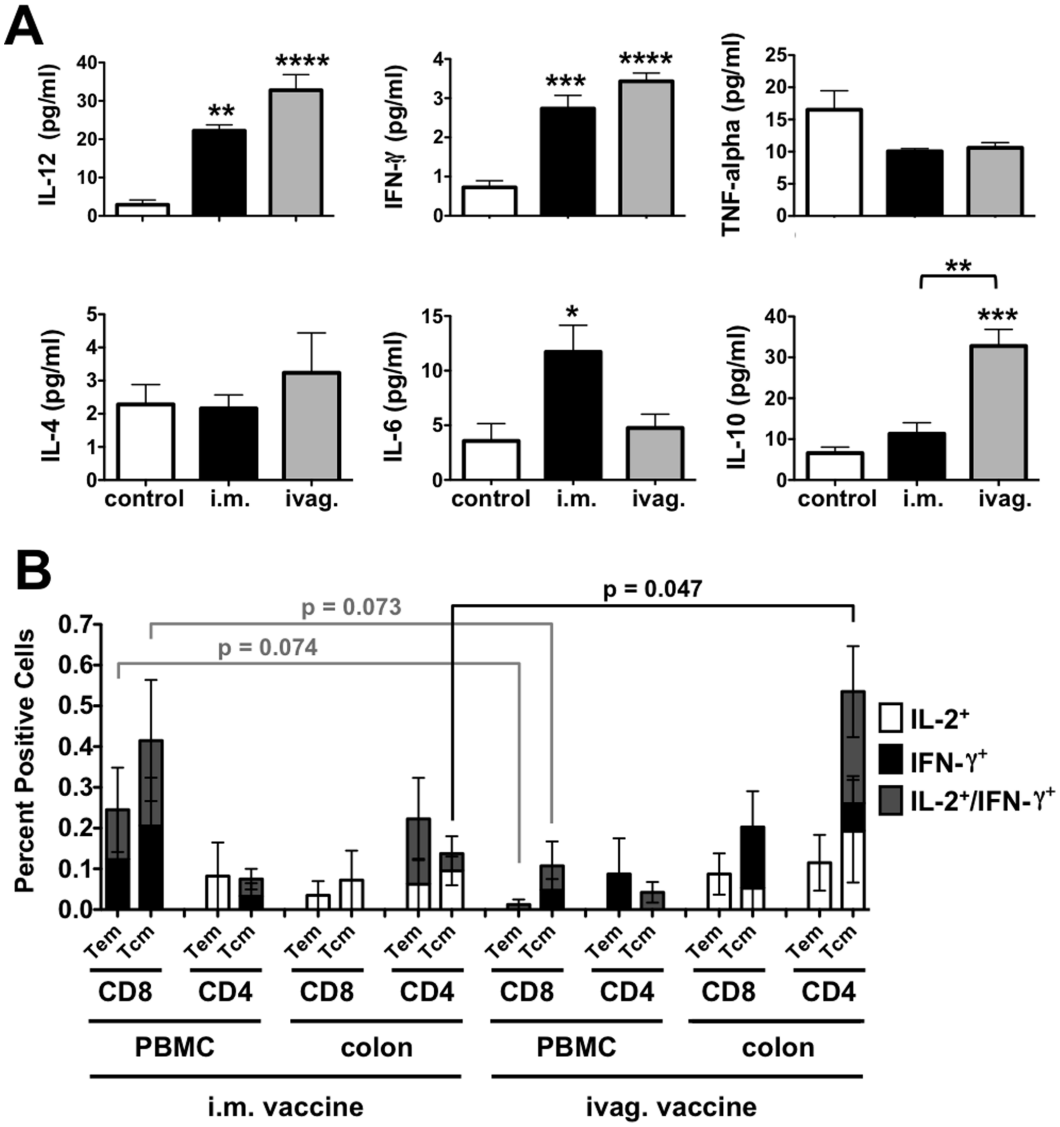
Systemic Cytokines, Peripheral and Mucosal T Cell Responses. **A**) Cytokine Responses. Heparinized plasma was collected and analyzed for cytokine levels using a human cytometric bead assay (CBA) with standard curves for each cytokine. Statistical comparisons in A are by one way ANOVA. **B**) T cell responses from PBMCs and colon biopsies. PBMCs and colon biopsies were collected at week 26 before challenge and CD4 and CD8 Tem and Tcm were analyzed for IL-2 and/or IFN-γ production by flow cytometry (Table 1). Statistical comparisons in B are T test comparisons of the "stack" of total cytokine-expressing cells for a given cell type (i.e. CD8 or CD4, Tcm or Tem).

Plasma cytokine levels of control and immunized animals were measured at week 26 after vaccination, but before challenge (Fig. 4A). Vaccination by both routes increased plasma IL-12 and IFN-γ levels significantly over levels in control animals (p<0.01 by one way ANOVA). IL-6 levels were significantly higher in the i.m. group than in controls (p<0.05), but not than in the ivag. group. In contrast, IL-10 levels were significantly higher in the ivag. group than control animals (p<0.001). IL-10 levels in the ivag. group were also significantly higher than in the i.m. group (p<0.01).

### Systemic and Mucosal Effector Memory and Central Memory T Cell Responses

At week 26, PBMCs and colon biopsies were collected for flow cytometry to analyze HIV-specific T cell compartments (Fig. 4B). Aliquots of 0.25 to 1×10^6^ cells from the single cell suspensions of PBMCs or colon biopsy samples were stimulated with HIV env peptide-pulsed DC as in [24] and stained with CD3, CD4, CD8, CD28, and CD95 for T cell subsets combined with IFN-γ and IL-2 to detect responses to HIV env. In the i.m. group, the strongest responses were observed in PBMC CD8 cells expressing IL-2 or IFN-γ in both the T cell effector memory (Tem) and T cell central memory (Tcm) compartments (Fig. 4B). In colon samples, the i.m. group generated detectable levels of CD4 and CD8 Tem and Tcm with stronger responses observed in CD4 cells. In contrast to the i.m. group, the ivag.-immunized animals generated weaker PBMC responses, but stronger responses in the colon. CD4 Tcm responses in the colon were particularly higher in the ivag. group. In this case, three of the four animals had dual functional IL-2/IFN-γ^+^ Tcm in the colon (H141, H225, and H245) and the fourth did not (animal 48). In contrast, only one animal in the i.m. group (animal 20) had IL-2/IFN-γ^+^ Tcm in the colon. Comparison of cytokine secreting cells in different compartments demonstrated significantly higher colon CD4/Tcm cytokine secreting cells in ivag.-immunized animals as compared to the same compartment in i.m.-immunized animals (p<0.05 by T test). Conversely, CD8 Tem and Tcm cytokine-secreting cells from PBMCs were higher in i.m.-immunized animals than in ivag.-immunized animals.

### Immunization by Species C HD-Ad Vectors during Multiple Rounds of Exposure to Species C Adenoviruses

Serotype-switching of Ad vaccines is an effective method to evade pre-existing and vector-induced immune responses [5], [7], [25], [26]. However, concerns have been raised that any adenovirus vector will stimulate cross-reactive T cell responses against conserved epitopes in the hexon protein that can blunt efficacy [27].

This HD-Ad vaccine study provides a unique model of the effects of immunity against adenoviruses of related serotypes. Ad1, 2, 5, and 6 are all species C adenoviruses with high amino acid identity for most of the proteins. Two exceptions to this are hexon and fiber proteins that have high serotype-specific variation. Because of this, serotype-switching is generally thought to evade antibodies primarily against hexon and to a lesser degree to evade antibodies against fiber.

In these experiments, macaques were immunized five times with four viruses from species C (Ad5, Ad6, Ad1, Ad5, and Ad2) and their ability to drive anti-HIV T cell responses were quantitated. After immunization with FG-Ad5, HD-Ad6 generated detectable anti-Env T cell responses (Fig. 2 and 3). After immunization with FG-Ad5, HD-Ad6, and HD-Ad1, HD-Ad5 itself generated robust T cell responses in PBMCs. Finally, HD-Ad2 generated robust T cell responses after four rounds of prior immunization with other species C adenoviruses. These data suggest that several rounds of exposure to adenoviruses from the same species does not prevent the generation of T cell responses against immunogens expressed by HD-Ad HIV vectors.

### Systemic and Mucosal Antibody Responses

**Figure 5:**
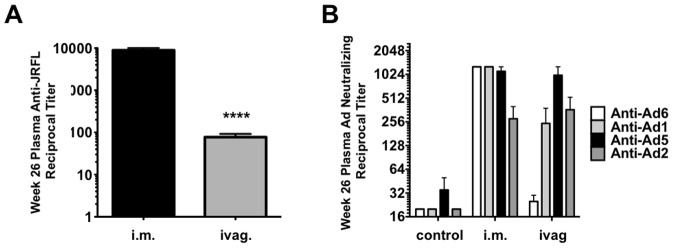
Antibodies Against HIV-1 Env and Species C Adenoviruses. **A**) Anti-env antibodies. Plasma was collected from each group and these were analyzed for env-binding antibodies by ELISA against JRFL env (matching the vaccine). **B**) Anti-adenovirus neutralizing antibodies. Plasma from week 26 was serially diluted and tested for its ability to neutralize HD-Ad vectors expressing luciferase *in vitro.*

Plasma and mucosal washes were collected two weeks after the last immunization and were assayed for HIV-1 envelope-binding antibodies by ELISA (Fig. 5). The i.m. group generated significantly higher anti- envelope antibody titers in the plasma than the ivag. group (Fig. 5A, p<0.0001). Plasma and vaginal washes were assayed for their ability to neutralize HIV-1 *in vitro.* No significant neutralizing antibody titers were detected in the plasma or vaginal washes after immunization as compared to the unimmunized controls (Figure S2). Plasma PBMCs were assayed for antibody-dependent cellular cytotoxicity (ADCC), but none of this activity was observed. These data suggest that despite env-binding antibody production by the two vaccine routes, no other antibody correlates were observed. This is consistent with previous experiments where multiple boosts with HD-Ad-Env generated robust ELISA responses [12], but not neutralizing antibodies [10].

Recent data has suggested a possible correlation between protection and variable loop binding antibodies in the RV144 trial [28]. To test if similar binding antibodies might be present in the vaccinated macaques, ELISAs against JRFL env were performed in the presence or absence of competing 15-mer peptides representing the variable loop domains of env. Under these conditions, none of the variable peptides blocked binding of macaque plasma antibodies to env (Figure S2).

### Anti-adenovirus Antibodies

Plasma samples at 26 weeks were analyzed for neutralizing antibodies against Ad1, Ad2, Ad5, and Ad6 after immunizations with FG-Ad5, HD-Ad6, HD-Ad1, HD-Ad5, and HD-Ad2 (Fig. 5B). At this time point, i.m. immunizations neutralizing titers over 1,000 against Ad6, 1, and 5, with 4-fold lower antibodies against Ad2, the last vaccine utilized. In contrast, samples from the vaginally-immunized animals had highest antibody levels against Ad5, with intermediate levels against Ad1 and Ad2, and near background levels of anti-Ad6 activity.

### Mucosal Challenge with CCR5-Tropic SHIV-162P3

**Figure 6:**
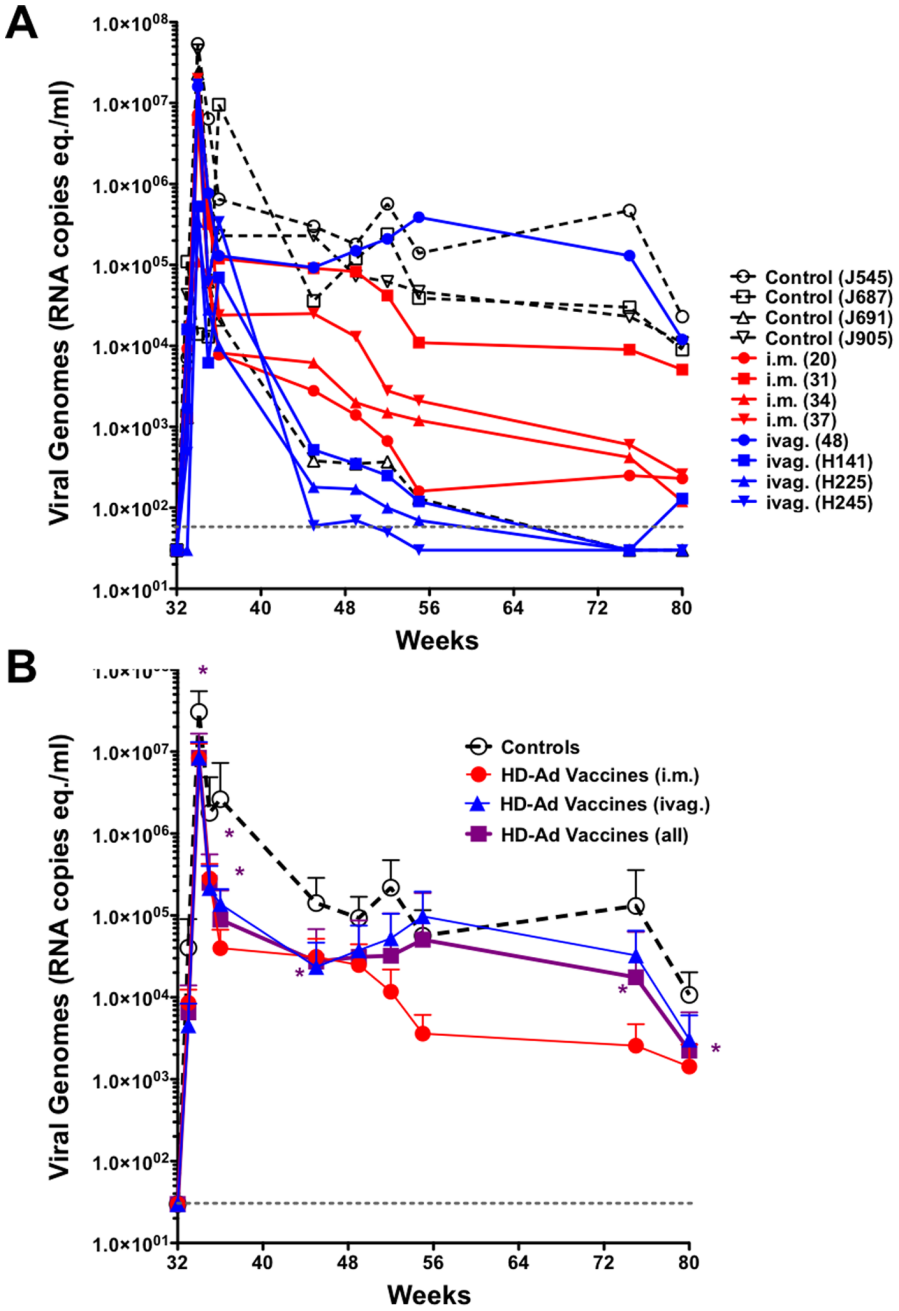
SHIV Viremia After Rectal Challenge. **A**) SHIV viral genomes were quantitated from plasma at the indicated times after challenge for each animal. The reported minimal detection for this assay of 30 meq./ml is indicated by a dashed gray line. **B**) Comparison of mean SHIV viral loads for controls, i.m. vaccinated, ivag. vaccinated, and the combined mean for all vaccinated animals. Asterisks indicate time points at which p<0.05 for controls as compared to combined vaccinated animals by T test. I.m. and ivag. only groups did not reach this p value.

Eight weeks after the last HD-Ad immunization, the animals were challenged by the rectal route with 1000 TCID50 of SHIV-162P3 and viral loads were monitored for 58 weeks after challenge. Three of four control animals maintained viral set points above 10^4^ copies/ml (Fig. 6A). The exception was control animal J691 whose viremia fell over 24 weeks to low levels. Spontaneous control was not observed in controls in our previous study [10], however, other studies have shown that SHIV SF162P3 can be spontaneously controlled in approximately 25% of unvaccinated animals [22]. Three of four i.m. immunized macaques had lower viral set points than most controls, with one (animal 31) having levels above 10^4^ copies/ml. In the ivag. group, three of the macaques had viral loads that fell precipitously over 20 weeks after challenge and fell at times below the level of detection. One ivag. group macaque had high viral loads above 10^4^ copies/ml that were comparable to most of the controls.

When the viral loads between control and immunized animals were compared by one way ANOVA, p values remained above 0.05 due in part due to spontaneous control of viremia in control animal J691. One-tailed T test for viremia 2 weeks after challenge at week 34 between control vs. i.m.-immunized animals gave p  = 0.067 and p  = 0.070 for control vs. ivag. animals. T test for viremia at week 55 between control vs. i.m.-immunized animals gave p  = 0.063 and p  = 0.35 for control vs. ivag. animals. When they were compared at week 80, the p value was 0.051 for control vs. i.m.-immunized animals and was 0.11 for control vs. ivag. animals. These data suggest that comparison with larger groups of animals might reach better statistical significance.

The animals remained healthy over the 90 weeks of the experiment. Follow up after 90 weeks showed that the animals remained healthy with the exceptions of animal #48 from ivag. group and J545 from control group. These animals had to be euthanized due to AIDS-like symptoms.

At the end of the study, the macaques were genotyped for major histocompatibility complex (MHC) alleles (Table 1). MHC alleles were varied within the animals and no overt association was observed between each animal's genotype and control of SHIV.

**Table 1:** Mamu MHC Genotypes in Study Animals.

Animal	A01	A02	A08	A11	B01	B03	B04	B08	B17	B29
J545	−	−	−	+	−	−	−	−	−	−
J687	−	−	+	+	+	−	−	−	−	−
J691	−	−	+	+	−	−	−	−	+	+
J905	−	−	+	+	−	−	−	−	−	−
Rh20	−	+	−	−	−	−	−	−	−	−
Rh31	−	−	+	+	+	−	−	−	−	−
Rh34	−	−	+	+	−	−	−	−	+	+
Rh37	−	+	−	+	+	−	−	−	−	−
Rh48	−	−	+	−	−	−	−	−	−	−
H141	−	−	+	−	−	−	−	−	+	+
H225	−	+	−	−	−	−	−	−	−	−
H245	−	+	+	−	−	−	−	−	−	−

### Individual Responses to Immunization and Challenge

After last immunization at week 26, no neutralizing or ADCC antibody responses were observed in the plasma or vaginal washes of the animals (Figure 2 and data not shown). 100-fold higher env-binding antibody titers were observed in plasma from the i.m. group when compared to the ivag. group (Fig. 5A). However, three of the four animals in the ivag. group have lower viremia than the i.m. group, suggesting that env binding antibodies were not associated with reductions in viral load.

Thirteen weeks after challenge (week 45), cellular immune responses in controls were two to three-fold lower than in the i.m. and ivag. groups (Fig. 2 and 3). Over the next 12 weeks, most of the control and vaccinated animals mounted increasing levels of IFN-γ responses. For 50 weeks after challenge, most IFN-γ responses declined, but a few animals' responses increased later. In contrast to IFN-γ ELISPOT responses from PBMCs, perforin ELISPOT responses from these cells showed low magnitude in all of the animals after challenge with some increases for animals 31 and 34 in the i.m. group 50 weeks after challenge.

Control animals J545, J687, and J905 had high plasma viremia levels, whereas J691's levels were lower (Fig. 2B). All but J687 had detectable IFN-γ responses in PBMCs after challenge, but these did not appear to track with high or low viral loads.

The i.m. immunized animals had significant levels of anti-HIV cellular immunity in PBMCs by ELISPOT prior to the virus challenge (Fig. 2 and 3). SHIV challenge boosted these anti-HIV cellular responses. The ivag. immunized animals that showed the best control over plasma viremia, H225 and H245, had the highest levels of anti-HIV IFN-γ responses at week 44, 12 weeks after challenge (Fig. 2A). The outlier in this group animal 48 that did not control the virus, had similar anti-HIV IFN-γ responses before challenge as compared to the other animals. However, animal 48's anti-HIV IFN-γ responses after challenge remained lower than the other animals in this group.

The i.m. immunized animals had the highest numbers of cytokine-secreting CD8 Tem and Tcm in PBMCs (Fig. 4B). In contrast, ivag. animals had low numbers of these cells, yet controlled virus as well or better than the i.m.-immunized animals suggesting that these cells may not be most associated with viral control. Three of the four intravaginally-immunized macaques had detectable dual functional IL-2/IFN-γ^+/+^ CD4 Tcm in the colon (H141, H225, and H245) 6 weeks before challenge, but animal 48 that did not control viremia did not have these responses (Fig. 2A and B). Animals H141, H225, and H245 had the lowest viremia of all macaques in the study whereas animal 48 had high levels of virus (Fig. 2B). Within the i.m. group, only animal 20 had detectable IL-2/IFN-γ^+/+^ Tcm in the colon. Animal 20 also had the lowest viremia in the i.m. immunized group. Taken together, these three ivag. and one i.m. immunized animals had both the lowest viremia after mucosal challenge and also had the highest IL-2/IFN-γ^+/+^ CD4 Tcm responses in the colon of all the animals.

## Discussion

This study was intended to compare systemic versus mucosal vaccines for their abilities to repel mucosal challenge by SHIV. We compared systemic (i.m.) vaccination with mucosal (ivag.) vaccination with HD-Ad vectors in the rhesus macaque model. We modeled a scenario of prior immunity to Ad5 by immunizing macaques with Ad5 before vaccination. The macaques were then immunized by the i.m. or ivag. route with HD-Ads expressing only the HIV envelope immunogen. The animals were immunized four times by the two routes with serotype switching of species C HD-Ads, 6, 1, 5, and 2 prior to challenge by the rectal route.

One caveat to this comparison is that we altered the vaginal delivery method at immunization three from lavage to cervical epithelial injection because of concerns with how well the vaccines were “taking”. While the method changed, we believe this shallow injection into the cervix maintains the “mucosal route”, since the injection is restricted to this locale. One can draw a parallel between this and a natural HIV infection where the vaginal epithelial is also thought to have small breaks that absorb incoming virus. While we cannot formally exclude that Ad applied by this method might not leak beyond the mucosal site, the qualitatively different immune responses that were observed suggest different route effects were maintained.

Both routes of immunization induced elevated IL-12 and IFN-γ cytokines in the blood of the animals to similar levels that are thought to support T_H1_ cellular immune responses. The i.m. route produced stronger IL-6 levels than controls while the ivag. route generated stronger IL-10 levels than controls and the i.m. group. IL-6 is an inflammatory cytokine that is also strongly associated with early innate immune responses to many infections, but also against adenoviruses [29]. IL-10 is generally thought to bias responses towards T_H2_, but may also be associated with T_reg_ responses.

Neither route generated antibodies with neutralizing, ADCC activities. ADCC was measured using whole PBMCs, so it is possible that stronger responses might be detected on purified NK cells. We were unable to detect V1/V2 binding antibodies as were observed in the RV144 trial [28] in our samples. Both routes of immunization produced envelope-binding antibodies in the plasma as measured by ELISA. However, the i.m. route generated plasma titers that were 100-fold higher than those generated by the ivag. route, yet more animals in the ivag. group had lower viral loads than in the i.m. group.

As expected, T cell responses from PBMCs were generally stronger after i.m. immunization than by ivag. immunization before challenge. These T cell responses climbed to higher frequencies after challenge than in control animals suggesting that vaccine groups were undergoing robust anamnestic recall responses. Of these, the ivag. group surprisingly had slightly higher responses relative to the i.m. group when compared to controls.

When cytokine production was assessed in Tem and Tcm cells from PBMCs and from colon biopsies before challenge, marked differences between the groups were observed. The i.m. route generated more robust IL-2 and IFN-γ responses in PBMC CD8 Tem and Tcm consistent with ELISPOT data. In contrast, CD8 responses in the colon samples were somewhat stronger in the ivag. group than the i.m. route group. CD4 responses were infrequent from PBMCs, but were more frequent for both groups in the colon samples. All four animals in the ivag. group generated CD4 Tcm cells in the colon. Three of the four i.m. group animals generated Tcm cells, but these were all at lower percent positive than all of the ivag. samples. When considering dual function IL-2/IFN-γ^+/+^ CD4 Tcm cells, three of four ivag. animals generated these central memory cells in the colon, whereas only one i.m. group animal generated these cells. Higher IL-2/IFN-γ^+/+^ CD4 Tcm cells correlated negatively with viral loads at weeks 55 and 80, suggesting them play a role in controlling viremia after mucosal infection by SHIV.

These data observing stronger PBMC T cell responses after i.m. immunization and better T cell responses in the colon after ivag. immunization are consistent with the hypothesis that systemic immunization drives better systemic immune responses and mucosal immunization drives better mucosal immune responses. Systemic and mucosal vaccination both generated immune correlates, with some indication that the ivag. route of immunization generated better protection against challenge in this small set of animals. These data support additional studies to determine if mucosal immunization does indeed have advantages when vaccinating against the mucosal pathogen HIV-1.

## Materials and Methods

### Adenoviruses

HD-Ad1, 2, 5, and 6 viruses expressing the gp140 form of HIV-1 JRFL were produced as previously described [12]. Briefly, HD-Ad5-env vector was transfected into a 60-mm dish of Cre-expressing 116 cells expressing Cre recombinase as in [30]. The transfected cells were infected a day later with the E1-deleted Ad5 helper virus AdNG163 whose packaging signal is flanked by loxP sites [30] for deletion in the Cre cells. Lysates were subsequently amplified by serial infections with AdNG163 in 116 cells. CsCl-banded HD-Ad were then produced from 3 liters of 116 cells producing HD-Ad preps with E1 -deleted helper contamination less than 0.02% [30]. HD-Ad1, 2, and 6 vectors were generated with helper viruses Ad1LC8cCEVS-1, Ad2LC8cCARP [31], and Ad6LC8cCEVS-6, respectively that were generously provided by Carole Evelegh and Frank L. Graham (McMaster University).

### Animals

All animal experiments were approved by the Institutional Animal Care and Use Committee at the University of Texas MD Anderson Cancer Center and were carried out according to the provisions of the Animal Welfare Act, PHS Animal Welfare Policy, and the principles of the NIH Guide for the Care and Use of Laboratory Animals, and the policies and procedures of the University of Texas MD Anderson Cancer Center. Twelve adult female rhesus macaques (*Macaca mulatta*) of Indian origin were maintained in the specific pathogen-free breeding colony at the Michael Keeling Center for Comparative Medicine and Research of The University of Texas MD Anderson Cancer Center, Bastrop TX. The chamber size for the animals was 44′W × 88′H × 160’D. All monkeys were given water ad libitum and were fed a commercial monkey diet (Harlan). Additional enrichment is provided in the form of manipulanda, visual stimulation or auditory stimulation and combinations thereof. Animals were monitored daily, including weekends and holidays. Anesthetics/analgesics were used to minimize any discomfort, distress, pain, and injury the animal might experience. As an endpoint, animals were euthanized with ketamine (11 mg/kg) followed by Beuthanasia (1 ml/10 lb)]. If any animal was moribund, unresponsive to treatment, could not eat or drink, was severely sick, or had symptoms of SAIDS, it was euthanized as per guidelines. The animals were anesthetized during procedures to minimize discomfort. Human PBMCs were obtained under approval of the Duke University Health System Institutional Review Board. Written informed consent was provided by study participants and/or their legal guardians.

### Induction of Pre-existing Anti-Ad5 Immunity

Groups of four macaques were used in these experiments. The macaques were sedated and immunized intranasally with 1×10^11^ vp of Adenovirus serotype 5 expressing β-galactosidase (Ad-LacZ). Blood, nasal lavages, vaginal lavages and colon biopsies were collected pre-immunization.

### Systemic and Mucosal HD-Ad-Env Immunization

Macaques were immunized intramuscularly (i.m.) or intravaginally (ivag.) with 10^11^ vp of HD-Ad6-JRFL, HD-Ad1-JRFL, HD-Ad5-JRFL, and HD-Ad2-JRFL at weeks 4, 7, 15 and 24, respectively (Fig. 1). The first two intravaginal immunizations involved simple application of 0.5 ml of the virus solution in PBS onto the vaginal epithelium. Because of concerns that this might not mediate robust transduction [32], the intravaginal immunizations with HD-Ad5-JRFL and HD-Ad2-JRFL on weeks 15 and 24 were performed by injection into the vaginal wall near the cervix using a 20 gauge catheter guided by an Otoscope.

### Collection of Samples

Samples were collected at each time point indicated before any immunization or procedure. Peripheral venous blood samples were collected in EDTA or sodium heparin. Before the separation of peripheral blood mononuclear cells (PBMC) from the blood samples, plasma was separated and stored immediately at -80°C. Peripheral Blood Mononuclear Cells (PBMCs) were prepared from the blood on Ficoll-Hypaque density-gradients.

### Colon Biopsies

The macaques were fasted for a minimum of 12 hours prior to the having colon biopsy to evacuate the stomach, duodenal, and proximal jejunal contents. They were then anesthetized with 3–5 mg/kg of Telazol, and placed in left lateral recumbency with a mouth guard to protect the endoscope. Using a human pediatric flexible endoscope with up to 7.8 mm in diameter, the gastroscopy was accomplished by advancing the endoscope through the colon and 1 to 2 mm biopsies were performed at the desired sites with up to 10–15 biopsies at each time point. In this procedure the monkey were given penicillin post biopsy for one day and 0.005 to 0.01 mg/kg Buprenorphine for 2 days. The monkey’s health, appetite, and stool were monitored twice per day.

### Isolation of Lymphocytes from Colon Biopsies

Colon biopsy specimens were washed once with cold PBS and the tissues were incubated at 37°C in RPMI 1640 containing 5% fetal bovine serum supplemented with type IV collagenase (Sigma-Aldrich) at 300 U/ml and DNase I at 15ug/ml with gentle shaking. The released cells were filtered through a 70 µm nylon mesh screen. Lymphocytes were purified on Percoll gradients (35–65%) at 1,500 RPM for 25 min. Lymphocytes were collected above 65% Percoll layers and washed two times before processing for staining.

### Collection of Mucosal Washes

Nasal secretions were collected using a phosphate buffered saline moistened sterile swab. The swab was placed in the nose, rotated and the swab was incubated in a collection tube with 1 ml of cold PBS to release the sample. Vaginal secretions were collected by lavage of the vagina with 10 ml of PBS with a 10 ml syringe.

### ELISA

To measure anti-Env plate binding antibodies, ELISAs were performed on macaque plasma and nasal washes after the last immunization as previously described [24]. Briefly, Immulon 4 HBX plates (Thermo, Milford, MA) were coated with 100 µl of HIV-1 envelope (Env) protein, JRFL-gp140CF and SF162 gp120 (NIH AIDS Reagent and Repository) at 1 µg/ml in PBS for 2 hours at room temperature (RT). The plates were blocked for 1 h with 1.0% BSA for 1 hour. Plasma and nasal washes were diluted 1∶100 and 1∶4, respectively, in PBS with 1.0% BSA and added to the plate for 1 h at RT. The plates were washed with 5 times PBS and 100 µl of Goat anti-monkey (H&L) HRP conjugated antibody (Pierce, Rockford, IL) diluted 1∶2500 in PBS with 1.0% BSA was added to the plate for 1 h at RT. The plates were washed 5 times with PBS and 100 µl of 1 Step Ultra TMB-ELISA substrate (Pierce, Rockford, IL) was added for 1 h at RT. The reaction was stopped with 50 µl of 2 M sulfuric acid and analyzed at 450 nm using a Beckman Coulter DTX 880 Multimode Detector.

### Assay for Neutralization of HIV and SHIV

Neutralization was measured as a reduction in luciferase reporter gene expression after a single round of infection in TZM-bl cells as described [33], [34]. TZM-bl cells were obtained from the NIH AIDS Research and Reference Reagent Program, as contributed by John Kappes and Xiaoyun Wu. Briefly, 200 TCID_50_ of virus was incubated with serial 3-fold dilutions of test sample in duplicate in a total volume of 150 µl for 1 hour at 37°C in 96-well flat-bottom culture plates. Freshly trypsinized cells (10,000 cells in 100 µl of growth medium containing 75 µg/ml DEAE dextran) were added to each well. One set of control wells received cells+virus (virus control) and another set received cells only (background control). After a 48 hour incubation, 100 µl of cells was transferred to a 96-well black solid plates (Costar) for measurements of luminescence using the Britelite Luminescence Reporter Gene Assay System (PerkinElmer Life Sciences). Neutralization titers are the dilution at which relative luminescence units (RLU) were reduced by 50% compared to virus control wells after subtraction of background RLUs. Assay stocks of molecularly cloned Env-pseudotyped viruses were prepared by transfection in 293T cells and were titrated in TZM-bl cells as described [33]. The clade B reference Env clones were described previously [33].

### Antibody Dependent Cellular Cytotoxicity (ADCC)

The presence of ADCC-mediating antibody responses was measured using PBMCs as previously reported using the flow-based GTL ADCC assay [35]. The testing of samples was conducted using SF162 recombinant gp120 (GeneBank No. AAT67508; ImmuneTechnology, Corp) to coat CEM.NKR.CCR5 target cells. PBMC from a normal human donor were utilized as source of effector cells. The results were analyzed to determine the maximum ADCC activity and the titer of ADCC Ab. The cut off for positive responses was set at 8% GTL activity.

### Cytokine Measurements

Non-Human Primate Cytokine kit with IL-2, IL-4, IL-6, IL-10, IL-12/23(p40), IFN-γ and TNF-α was purchased from Millipore Corporation (Billerica, MA). Plasma concentrations of cytokines were determined according to the manufacturers’ protocols. Multianalyte profiling was performed on the Bio-Plex 200 system and the micro plate Platform (Luminex X MAP technology). Calibration microspheres for classification and reporter readings as well as sheath fluid were also purchased from Millipore Corporation. Acquired fluorescence data were analyzed by the Bio-Plex manager 5.0.

### ELISPOT Assay For Detecting Antigen-Specific IFN-γ And Perforin Producing Cells

Freshly prepared PBMC were used for the IFN-γ or perforin ELISPOT assay as described previously [36]. PBMCs were either stimulated with synthetic peptides pools, with Ad expressing env, or with Con A (5 µg/ml) as positive control reagent. The SF162P3 overlapping env 15-mer peptide set (NIH AIDS Reagent Program) was used as 3 pools of 50 to 70 peptides spanning the gp140 region. PBMCs (1 x 10^5^) were seeded in duplicate wells of 96-well plates (polyvinylidene difluoride backed plates, MAIP S 45, Millipore, Bedford, MA) coated with anti-IFN-γ or anti-perforin. The cells were incubated in the presence of the various antigens for 36 h at 37°C. The cells were then removed, the wells washed, and then incubated with 100 µl of ALP conjugated secondary anti-IFN-γ or streptavidin-ALP for perforin assay for 2 h at room temperature. Spots representing individual cells secreting IFN-γ or perforin were developed using BCIP/NBT substrate as per protocol provided by manufacturer. The plates were washed to stop development and the spots were counted by an independent agency (Zellnet Consulting, New Jersey, NJ). The responses in terms of IFN-γ or perforin spot forming cells (SFC) for 10^5^ total input PBMC were determined for individual monkeys after subtracting background values of cells cultured in the medium. The cut off value for determining the positive response in the assay is defined as a minimum of 10 spots that is twice the number observed in cells cultured in the medium. Data is represented as SFUs per 10^5^ PBMCs.

### Intracellular Cytokine Assays

PBMC or single cell suspensions of colon biopsies were incubated at 37°C in a 5% CO_2_ incubator for 6 h in the presence of RPMI 1640–10% FBS medium alone (unstimulated), a pool of 15-mer env peptides (5 µg/ml each peptide), or with PMA and ionomycin (5 µg/ml; Sigma-Aldrich) as a positive control. All cultures contained monensin (GolgiStop; BD Biosciences) as well as 1 ug/ml anti-CD49d (BD Biosciences). The cultured cells were stained with monoclonal antibodies (MAbs) specific for cell surface molecules (CD3, CD4, CD8, CD28, and CD95) and an Aqua to discriminate live from dead cells as in [37]. After fixation with Cytofix/Cytoperm solution (BD Biosciences), cells were permeabilized and stained with antibodies specific for IFN-γ, and IL-2. Labeled cells were fixed in 1.5% formaldehyde-PBS. Samples were collected on an LSR II instrument (BD Biosciences) and were analyzed using FlowJo software (Tree Star). Approximately 200,000 to 1,000,000 events were collected per sample (see Figure S3 for examples). The background level of cytokine staining varied from sample to sample but was typically <0.01% of the CD4+ T cells and <0.05% of the CD8+ T cells. Samples were considered positive when the percentage of cytokine-staining cells was at least twice that of the background. ICS assays were performed on PBMC and single cell suspensions from colon biopsies obtained on week 26.

### Intrarectal Exposure to SHIV

To evaluate for protective efficacy and immunological correlates, all monkeys were challenged by intrarectal inoculation of 1,000 TCID_50_ of SHIV-SF162P3 from the NIH AIDS Reagent Program. The monkeys were fasted for a minimum of 24 hours prior to exposure. The animals were anesthetized (10 mg/kg of body weight ketamine intramuscularly [i.m.] and 0.5 mg/kg xylazine i.m.) and were placed in a sternal position with the pelvis propped up at an approximately 45° angle. A lubricated infant feeding catheter was inserted gently into the rectum of the animal approximately 4 to 6 inches without causing any injury. First 5 ml of diluent (PBS) was gently flushed through the catheter, and then 0.5 ml of the virus was injected through the catheter, followed by a 0.5 ml flush with diluents. The animal was returned to its cage and kept tilted at 45^0^ angle until it fully recovered from anesthesia.

### Viral Load Determination

SHIV viral loads from the blood were determined by determining viral RNA copy numbers by real-time RT-PCR analyses. These assays were performed at the NIH Core Facility by Dr. Jeff Lifson's group. The threshold sensitivity of the assay is 30 viral RNA copy-equivalents/ml of plasma, and the inter-assay variation is <25% (coefficient of variation).

### Major Histocompatibility Typing of Macaques in the Study

After completion of the study, PBMCs from study macaques were evaluated for *Mamu* haplotypes by the AIDS Vaccine Research of Dr. David Watkins at the University of Miami. *Mamu-A01, A02, A08, A11, B01, B03, B04, B08, B17, and B29* alleles by PCR (PCR Experiment # 121211 Mayo Clinic (Dr. Barry)).

### Statistical Analyses

Data was evaluated by ANOVA and T TEST using GraphPad Prism 4 software and Microsoft Excel. P values ≤0.05 were considered statistically significant.

## Supporting Information

Figure S1 **Alignment of JRFL and SF162P3 Envelope Proteins.**
(TIFF)

Figure S2 **Anti-Env ELISA in the Presence of Competing Env Peptides.**
(TIFF)

Figure S3 **Flow cytometry for Tcm and Tem. A)** Gating strategy. **B)** Representative flow cytometry scatter plots.
(TIFF)
