# Supplementary figures and images for: Comparison of Systemic and Mucosal Immunization with Helper-Dependent Adenoviruses for Vaccination against Mucosal Challenge with SHIV
Source: PLoS One. 2013 Jul 3;8(7):e67574. doi: 10.1371/journal.pone.0067574 (PMC3701068; doi:10.1371/journal.pone.0067574)

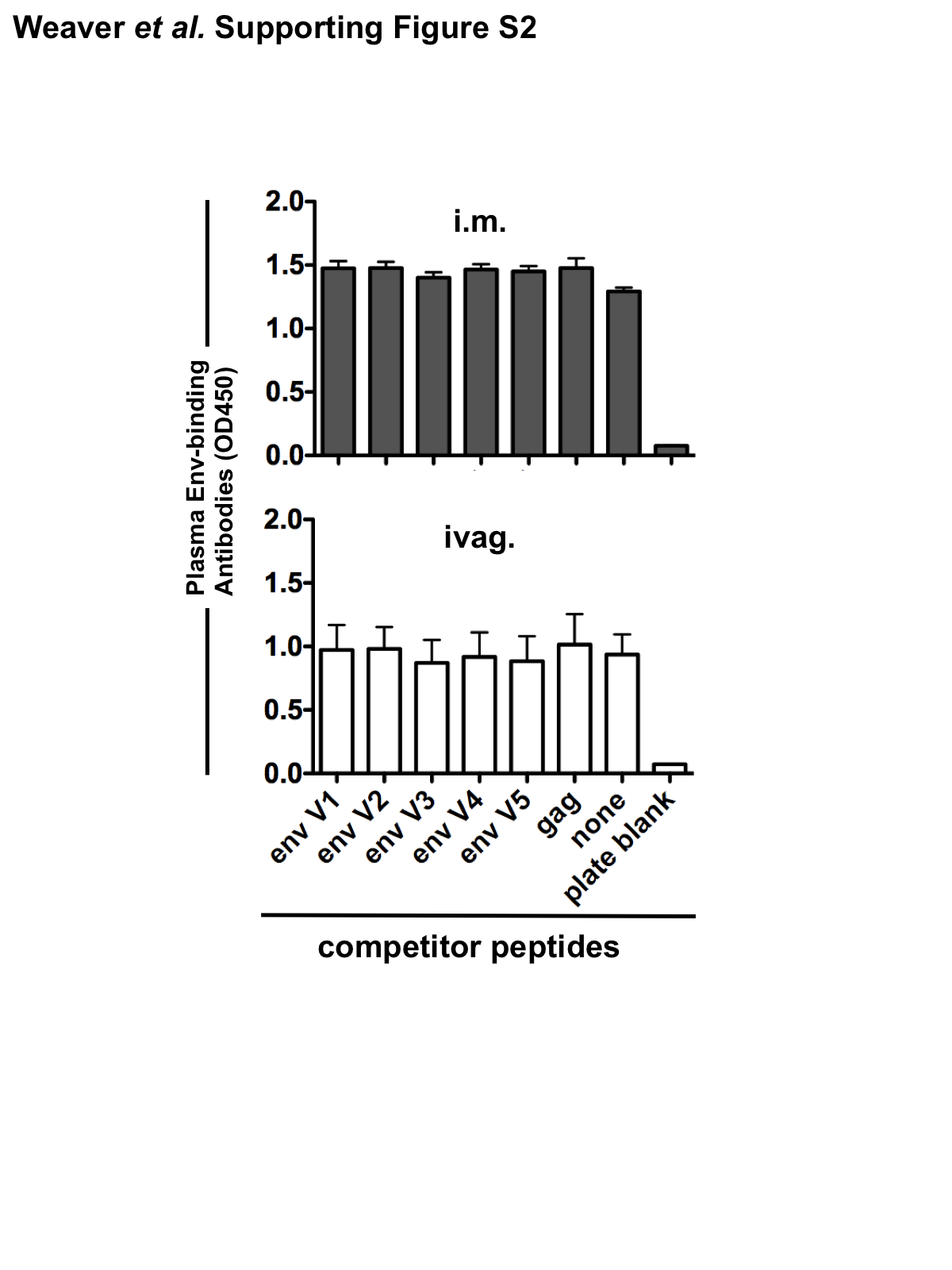

Supplement: Figure S2 — Anti-Env ELISA in the Presence of Competing Env Peptides. (TIFF) [file pone.0067574.s002.tiff]

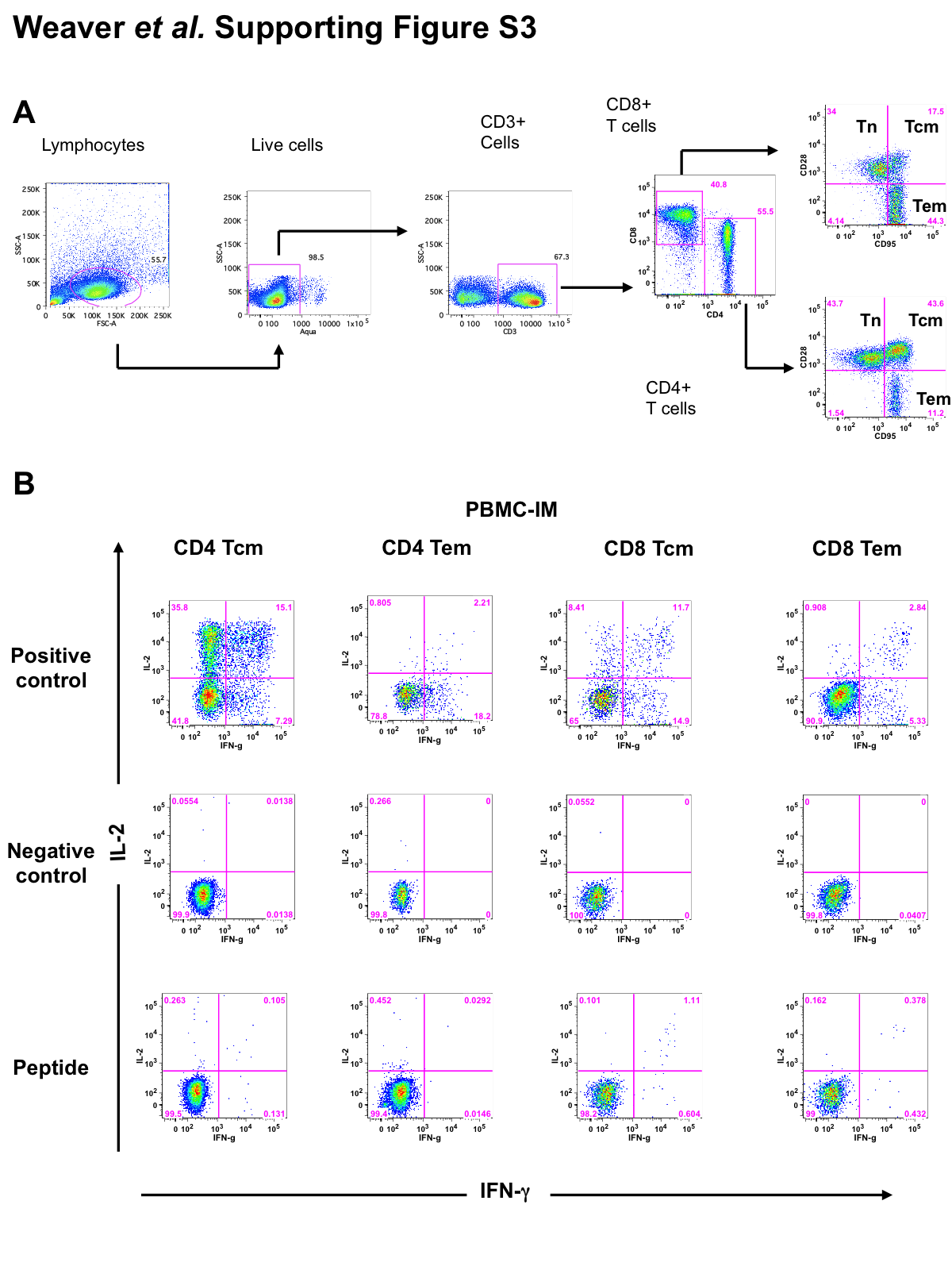

Supplement: Figure S3 — Flow cytometry for Tcm and Tem. A) Gating strategy. B) Representative flow cytometry scatter plots. (TIFF) [file pone.0067574.s003.tiff]
